# Altered functional connectivity of the amygdala and its subregions in typhoon‐related post‐traumatic stress disorder

**DOI:** 10.1002/brb3.1952

**Published:** 2020-11-18

**Authors:** Tao Liu, Jun Ke, Rongfeng Qi, Li Zhang, Zhiqiang Zhang, Qiang Xu, Yuan Zhong, Guangming Lu, Feng Chen

**Affiliations:** ^1^ Department of Neurology Hainan General Hospital (Hainan Hospital Affiliated to Hainan Medical College) Haikou Hainan Province China; ^2^ Department of Medical Imaging Jinling Hospital Medical School of Nanjing University Nanjing Jiangsu Province China; ^3^ Key Laboratory of Psychiatry and Mental Health of Hunan Province Mental Health Institute the Second Xiangya Hospital National Technology Institute of Psychiatry Central South University Changsha Hunan Province China; ^4^ School of Psychology Nanjing Normal University Nanjing Jiangsu Province China; ^5^ Department of Radiology Hainan General Hospital (Hainan Hospital Affiliated to Hainan Medical College) Haikou Hainan Province China

**Keywords:** amygdala, basolateral amygdala, functional connectivity, post‐traumatic stress disorder, trauma

## Abstract

**Background:**

New evidence suggests that the centromedial amygdala (CMA) and the basolateral amygdala (BLA) play different roles in threat processing. Our study aimed to investigate the effects of trauma and post‐traumatic stress disorder (PTSD) on the functional connectivity (FC) of the amygdala and its subregions.

**Methods:**

Twenty‐seven patients with typhoon‐related PTSD, 33 trauma‐exposed controls (TEC), and 30 healthy controls (HC) were scanned with a 3‐Tesla magnetic resonance imaging scanner. The FCs of the BLA, the CMA, and the amygdala as a whole were examined using a seed‐based approach, and then, the analysis of variance was used to compare the groups.

**Results:**

We demonstrated that the BLA had a stronger connectivity with the prefrontal cortices (PFCs) and angular gyrus in the PTSD group than in the TEC group. Additionally, compared with the PTSD and the HC groups, the TEC group exhibited decreased and increased BLA FC with the ventromedial PFC and postcentral gyrus (PoCG), respectively. Furthermore, the PTSD group showed abnormal FC between the salience network and default‐mode network, as well as the executive control network. Compared with the HC group, the TEC group and the PTSD group both showed decreased BLA FC with the superior temporal gyrus (STG). Finally, the FCs between the bilateral amygdala (as a whole) and the vmPFC, and between the BLA and the vmPFC have a negative correlation with the severity of PTSD.

**Conclusions:**

Decreased BLA‐vmPFC FC and increased BLA‐PoCG FC may reflect PTSD resilience factors. Trauma leads to decreased connectivity between the BLA and the STG, which could be further aggravated by PTSD.

## Graphical abstract

New evidence suggests that the centromedial amygdala (CMA) and the basolateral amygdala (BLA) play different roles in threat processing. Decreased BLA‐vmPFC FC and increased BLA‐PoCG FC may reflect PTSD resilience factors. Trauma leads to decreased connectivity between the BLA and the STG, which could be further aggravated by PTSD.

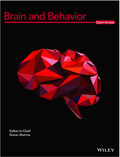

## 1 INTRODUCTION

Post‐traumatic stress disorder (PTSD) is a debilitating psychiatric disorder characterized by symptom clusters that may include re‐experiencing, avoidance, emotional numbness, and hyperarousal. However, the cerebral mechanisms involved in the appearance of PTSD symptoms and in PTSD pathophysiology remain unknown. Previous studies have used task‐based functional magnetic resonance imaging (fMRI) to delineate a pattern of hyperactivation in the amygdala, hypoactivation in the ventromedial prefrontal cortices (vmPFC), and aberrant function of the hippocampus (Acheson et al., 2012; Admon et al., 2013; Francati et al., 2007; Hughes & Shin, 2011; Pitman et al., 2012; Shin et al., 2006). Therefore, some scholars point to an underlying issue of damage to the neural circuits that regulate emotion in PTSD patients (the vmPFC–amygdala–hippocampal circuit model) (Acheson et al., 2012; Admon et al., 2013; Hughes & Shin, 2011; Rauch et al., 2006). A meta‐analysis of 15 neuroimaging studies on PTSD confirmed this pattern (amygdala hyperactivity and medial prefrontal cortex (mPFC) hypoactivity (Etkin & Wager, 2007)), which is generally thought to cause a lack of emotional control in PTSD patients.

The study of functional connectivity (FC) may offer additional information regarding the regulatory relationships between the amygdala and mPFC. The amygdala has close structural connections and mutual feedback loops with the dorsolateral PFC (Bracht et al., 2009), the mPFC, and the orbitofrontal cortex (Ghashghaei et al., 2007), in addition to the anterior cingulate cortex (ACC) (Robinson et al., 2010). High amygdala activity is correlated with lower mPFC activity in healthy individuals (Kim et al., 2011; Phan et al., 2002), and researchers have detected task‐based FC between these areas. Roy et al. (2009) reported correlations between the amygdala and vmPFC (including the rostral ACC and medial frontal gyrus), thalamus, insula, and striatum at rest; they also reported negative correlations with the posterior cingulate cortex (PCC), dorsal ACC, bilateral middle frontal gyrus (MFG), and the superior frontal gyrus, explained as functional dissociations between the affect‐production network and the emotion or cognitive‐adjustment network. Task‐related studies on the FC of the amygdalas of PTSD patients have yielded heterogeneous findings. For example, [^15^O]H_2_O positron emission tomography (PET) of individuals recently exposed to trauma has shown a positive correlation between the amygdala and ACC in response to traumatic scripts (Osuch et al., 2008). On the other hand, another PET study found a negative correlation between the amygdala and ACC during non‐trauma scripts and a decline in the intensity of this FC in PTSD patients (Gilboa et al., 2004). It is worth noting that, irrespective of the direction of the FC, most studies have detected a reduced connectivity strength in PTSD patients compared with controls (Fonzo et al., 2010; Osuch et al., 2008; Shin & Liberzon, 2010; Simmons et al., 2008). A rest‐state FC study can reduce the confounding task‐related factors that may be cause amygdala activity or trigger PTSD symptoms, which means it may be a better method to assess the connectivity. Rabinak et al. (2011) revealed enhanced amygdala–insula connectivity during resting state. Sripada et al. (2012) found positive FC between the amygdala and insula, decreased positive FC between the amygdala and hippocampus, and reduced negative FC between the amygdala and rostral ACC and dorsal ACC at rest. These data contradict the finding of declined FC during threat‐based processing (Fonzo et al., 2010).

One key limitation in previous studies exploring amygdala connectivity in PTSD patients has been the failure to use the amygdala subregions as a seed. The amygdala subregions include the basolateral amygdala (BLA) and the centromedial amygdala (CMA) nuclei, each of which has a distinct structure and FC (Brown et al., 2014; Etkin & Wager, 2007; Roy et al., 2009). The CMA, composed of the medial and central nuclei (including GABAergic neurons (Duvarci & Pare, 2014)), promotes behavioral reactions by increasing attention and motor agility via projections to the striatal regions (Davis, 1997; LeDoux, 2003; Roy et al., 2009), hypothalamus (which regulates cortisol release), brainstem, and basal forebrain (LeDoux, 1998). The BLA is composed of the basomedial, basoventral, lateral, and basolateral nuclei; its function is to integrate sensory input from the association cortex (Jovanovic & Ressler, 2010) and promote learning via thalamic projections (Brown et al., 2014; Etkin & Wager, 2007). New evidence suggests that the CMA and the BLA play different roles in threat processing (Fruhholz & Grandjean, 2013; Morris et al., 2001; Nicholson et al., 2015). Brown et al. (2014) found an increase in FC between the prefrontal cortical areas and the BLA related to affect regulation in PTSD patients compared with trauma‐exposed patients at rest. Furthermore, dissociative symptoms such as derealization and depersonalization in PTSD patients are correlated with increased FC between both the BLA and the CMA and prefrontal and cerebral areas involved in body proprioception and awareness at rest (including the dorsal posterior cingulum and precuneus) (Nicholson et al., 2015). Despite these suggestive findings, the FC of the amygdala subregions has yet to be fully explored in individuals exposed to typhoon‐related trauma. Are the changes specific to PTSD? How is FC changed in the trauma‐exposed controls (TEC)? What are the effects of different stressful events? All of these questions need to be further clarified.

Consequently, this study explores the differential contribution of the amygdala subdivisions to cerebral connectivity in PTSD patients, trauma‐exposed controls (TEC), and healthy controls (HC). In accordance with emerging reports, we anticipate finding differential FC between the CMA and BLA and other cerebral areas, particularly in reference to the BLA salience network and the BLA default‐mode network (DMN) in PTSD patients and TECs and their relationship with neuroplasticity and disease severity. We aimed to describe the role of each distinct FC alternation between the point of trauma exposure and the development of PTSD.

## 2 MATERIALS AND METHODS

### 2.1 Participants and clinical assessment

**Figure 1:**
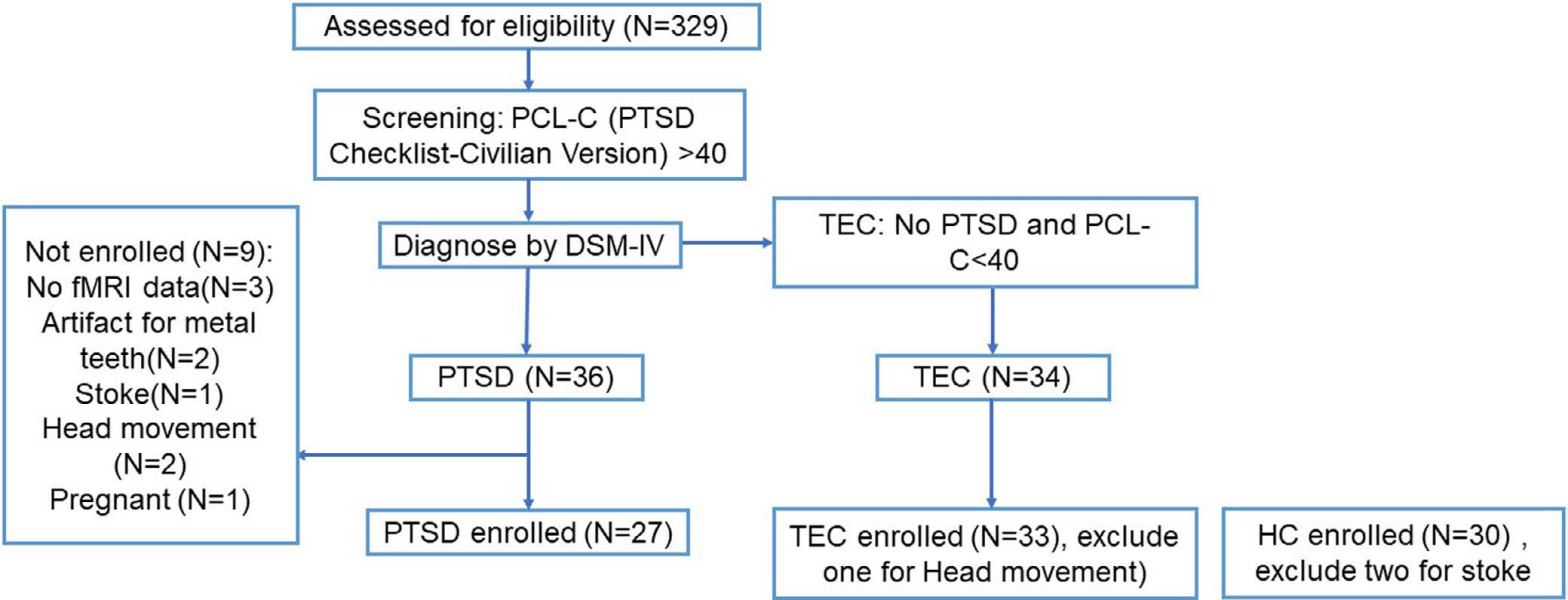
Flow diagram of study enrollment

On 18 July 2014, the category 5 super typhoon Rammasun struck the city of Wenchang in Hainan province, the island territory of China. Individuals living there were severely affected by this storm, which resulted in 14 deaths. In Luodou town, Wenchang city, more than 1,000 individuals were caught in the tide. Seventy individuals from this region exposed to the typhoon were enrolled in this study. Thirty‐six individuals were diagnosed with PTSD (nine male [M]/27 female [F]); 34 individuals were enrolled as TECs (M/F = 7/27) without PTSD. The PTSD Checklist—Civilian Version (PCL), a 17‐item self‐evaluation questionnaire that assesses the seriousness of DSM‐IV‐characterized PTSD manifestations on a 5‐point scale, was used to screen all subjects, and the enrollment diagram of the subjects is shown in Figure 1. Current PTSD was diagnosed according to the DSM‐IV criteria, and symptoms were evaluated using the Clinician‐Administered PTSD Scale (CAPS) (Weathers et al., 2001). CAPS uses a scale from 0 to 4 to evaluate the frequency and intensity of each manifestation of PTSD. Seventeen key symptoms of PTSD were surveyed; the detailed data included symptom onset, duration, and impact. All the patients were unmedicated before enrollment. The DSM‐IV Structural Clinical Interview was used to identify comorbid disorders. The HCs (M/F = 9/23) who were unable to meet the PTSD criterion were enrolled from Haikou city, about 35 km away from Wenchang. All participants were screened for anxiety and depression using the Self‐Rating Depression Scale (SDS) (Zung, 1965) and the Self‐Rating Anxiety Scale (SAS) (Zung, 1971). This procedure took 3 months, from November 2014 to January 2015.

The criteria for exclusion included the following: (a) <18 or >65 years of age; (b) those with a history of loss of consciousness or head injury; (c) those with significant medical and neurological conditions; (d) those with a history of or who currently exhibit signs of psychiatric disorders other than anxiety or depression, drug or alcohol abuse/dependence, and utilization of mental drugs; (e) left‐handedness; (f) contraindications for MRI, for example, pregnancy, ferromagnetic implants, or claustrophobia.

After MRI scanning, we excluded 9 PTSD participants for a lack of available imaging data (three female), artifacts related to dentures (one male and one female), cerebral infarction (one female), pregnancy (one female), and obvious movement during scanning (one male and one female). Moreover, one female in the TEC group was excluded for obvious movement during scanning, and two males in the HC group were excluded due to stroke. Finally, 27 PTSD participants, 33 TECs, and 30 HCs were included in the analysis.

The study was conducted in accordance with the declaration of Helsinki, and it was approved by the ethics committee of Hainan General Hospital and Second Xiangya Hospital of Central South University. All participants provided written informed consent after a detailed description of the study.

### 2.2 MRI data acquisition

The image data were acquired with a 3‐Tesla MRI scanner (MAGNETOM Tim Skyra; Siemens Medical Solutions) with a 32‐channel phased‐array head coil. High‐resolution T1‐weighted 3‐dimensional images were taken with a sagittal magnetization‐prepared rapid gradient‐echo sequence for later co‐registration and normalization (repetition time/echo time [TR/TE = 2,300/1.97 ms, flip angle = 9°, field of view [FOV] = 256 × 256 mm^2^, matrix = 256 × 256, slice thickness = 1 mm, 176 slices). Gradient‐echo planar imaging (EPI) was used to acquire blood–oxygen‐level‐dependent (BOLD) fMRI scans with an interleaved slice excitation order and a 2 mm isotropic spatial resolution (TR/TE = 2,000/30 ms, flip angle = 90°, FOV = 256 × 256 mm^2^, matrix = 64 × 64, slice thickness = 3.6 mm, 35 slices, no gap of intersection, total volume number = 250), scanning time 8 min and 28 s; the anterior commissure/posterior commissure line served as a parallel reference. During the rest‐state scans, all participants were asked to lie restfully, close their eyes, and let their thoughts wander without sleeping.

### 2.3 Data preprocessing

No subject with frame‐wise displacement (using the Jenkinson formula) >0.2 mm was found to have excessive head motion during MRI acquisition (Yan et al., 2013). The imaging data were processed using Statistical Parametric Mapping (SPM8, http://www.fil.ion.ucl.ac.uk/spm/). The initial 10 volumes of the functional images were removed to ensure signal equilibrium. The remaining 240 volumes were slice‐time corrected, realigned, and co‐registered with the anatomical scan. The co‐registered anatomical images were normalized into standard Montreal Neurological Institute (MNI) space for 3 × 3 × 3 mm^3^ after being divided into white matter (WM), gray matter (GM), and cerebrospinal fluid (CSF) (Spati et al., 2015). The resulting normalization matrix was then applied to the functional data. After that, the functional images were smoothed using convolution with an isotropic Gaussian kernel (full width at half‐maximum [FWHW] = 8 mm). After smoothing, the imaging data were filtered (bandpass, 0.01–0.08 Hz) to remove the effects of low‐frequency drift and high‐frequency noise. Finally, nuisance covariates, including cerebrospinal fluid signals, global mean signals, white matter signals, and head motion parameters, were regressed from the fMRI data. We used the head motion (HM) + its temporal derivatives and their squares method to remove head motion artifacts.

### 2.4 Seed‐based FC analysis

A region of interest (ROI)‐based inter‐regional FC analysis was conducted to investigate the functional connectivity between the amygdala and other areas of the brain. The bilateral BLA, CMA, and the amygdala as a whole (BLA + CMA) were selected as ROIs. The reslice size was 3 × 3 × 3 mm^3^. ROIs for seeds were created using SPM’s Anatomy Toolbox (Terada et al., 1998) with cytoarchitectonically based probability maps of the amygdala instantiated in the Juelich Brain Atlas (Uranishi et al., 1999). These seed regions were used to define the reference time series. Furthermore, a correlation map was generated after obtaining the cross‐correlation coefficient (*r* score) between the reference time series of each seed region and the rest of the brain. The Fisher Z‐transformation was performed to improve the normality of the correlation coefficient.

### 2.5 Statistical analysis

Statistical analysis was performed using SPSS 16.0 (SPSS Inc.). *p*‐Values less than 0.05 were considered statistically significant. The chi‐squared test was performed for gender distribution, and all continuous variables were analyzed using one‐way analysis of variance (ANOVA) except for PCL scores, for which the independent *t* test was used to examine the difference between the PTSD and the TEC groups.

SPM8 was used to analyze the FC maps of the three groups. For every group, a random‐effects one‐sample *t* test was used (*p* < .05, AlphaSim‐corrected). ANOVA was used to compare ROI‐based FC differences among the three groups with a depression diagnosis and education level as covariates, followed by a *t* test to examine between‐group differences if a statistical difference was noted (*p* < .001, cluster >10, uncorrected).

Correlation analysis between the mean FC (*Z* values) of the clusters, which showed a significant difference between groups in post hoc *t* tests and total CAPS scores, was conducted using Pearson's correlation coefficient.

## 3 RESULTS

### 3.1 Demographic and clinical variables

Clinical and demographic characteristics are summarized in Table 1. There was no significant difference in gender distribution (*p* = .912) or age (*F* = 0.317, *p* = .729) among the PTSD, TEC, and HC groups. The education level of the HC group was higher than the PTSD and TEC groups (*F* = 8.396, *p* < .001). The mean total CAPS score of the PTSD group was 78.2 ± 19.3. The PCL scores were higher in the PTSD group than that in the TEC group (*p* < .001). There were significant differences in the SDS (*F* = 101.915, *p* < .001) and SAS (*F* = 81.864, *p* < .001) scores of the three groups. According to SDS and SAS scores, there were nine participants (two males and seven females) with comorbid depression (SAS = 60.9 ± 6.0, 49–68; SDS = 65.6 ± 4.2, 58–72) and one female with comorbid anxiety (SAS = 62, SDS = 62) in the PTSD group. The SAS and SDS scores in the TEC group were significantly higher than those in the HC group, but they were significantly lower than the PTSD group.

**Table 1:** Demographic and clinical data of traumatized individuals and healthy controls

	PTSD (*n* = 27)	TEC (*n* = 33)	HC (*n* = 30)	*p* Value
Gender (males/females)	7/20	7/26	7/23	.912*
Age (year)	48.4 ± 10.3	48.5 ± 7.5	49.9 ± 6.1	.729**
Education (year)	6.4 ± 3.4	7.0 ± 3.4	9.7 ± 3.3	<.001**
Days after the disaster to examination	105.5 ± 9.5	118.0 ± 10.0	125.8 ± 1.0	<.001**
SAS score	65.8 ± 13.3	41.3 ± 8.1	36.0 ± 5.5	<.001**
SDS score	69.6 ± 13.2	41.3 ± 9.1	33.5 ± 7.2	<.001**
PCL score	53.7 ± 8.5	28.9 ± 5.4		<.001***
CAPS total score	78.2 ± 19.3			

Values are given as mean ± *SD* except for gender, which is presented as a number.

Abbreviations: CAPS, Clinician‐Administered PTSD Scale; HC, healthy control; PCL, PTSD Checklist; PTSD, post‐traumatic stress disorder; SAS, Self‐Rating Anxiety Scale; SDS, Self‐Rating Depression Scale; TEC, trauma‐exposed control.

* *P* value obtained with chi‐square test.

** *P* value obtained with one‐way analysis of variance.

*** *P* value obtained with independent *t* test for continuous variables.

### 3.2 One‐sample FC within the HC, TEC, and PTSD groups

**Figure 2:**
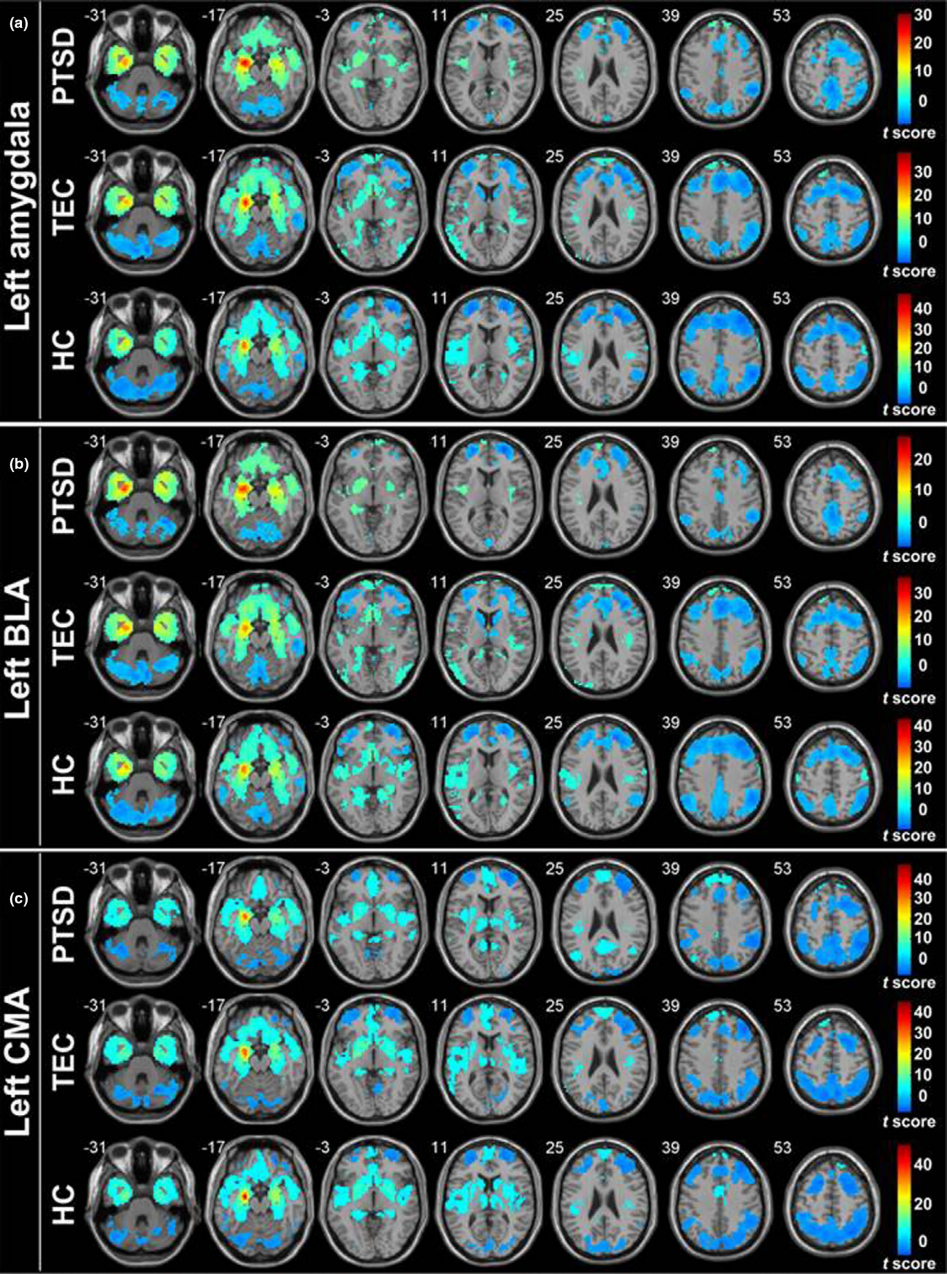
Functional connectivity of the left amygdala as a whole (a), BLA (b), and CMA with the whole brain (one‐sample*t*test,*p* < .05, AlphaSim‐corrected). Warm color represents the positive functional connectivity; cold color represents the negative functional connectivity.*Note*: BLA, basolateral amygdala; CMA, centromedial amygdala; HC, healthy control group; PTSD, post‐traumatic stress disorder group; TEC, trauma‐exposed control group

When the amygdala as a whole, BLA and CMA were selected as ROIs, the FC change modes of bilateral ROIs were similar, and the distribution of FC in all ROIs was nearly symmetrical, which revealed positive FC with the mPFC, ventral ACC, hippocampus, parahippocampal gyrus, fusiform gyrus, insula, and superior temporal gyrus (STG) within the HC, TEC, and PTSD groups. Conversely, the MFG, superior frontal gyrus, inferior frontal lobe, and the inferior parietal lobule/angular gyrus showed negative FC. Moreover, the BLA revealed positive FC with the precentral/postcentral gyrus (PoCG), and CMA revealed negative FC with the thalamus, putamen, caudate nucleus, and dorsal ACC. The FC results of left ROIs in each group are shown in Figure 2a–c (*p* < .05, AlphaSim‐corrected).

### 3.3 Group differences in whole amygdala resting‐state FC

**Figure 3:**
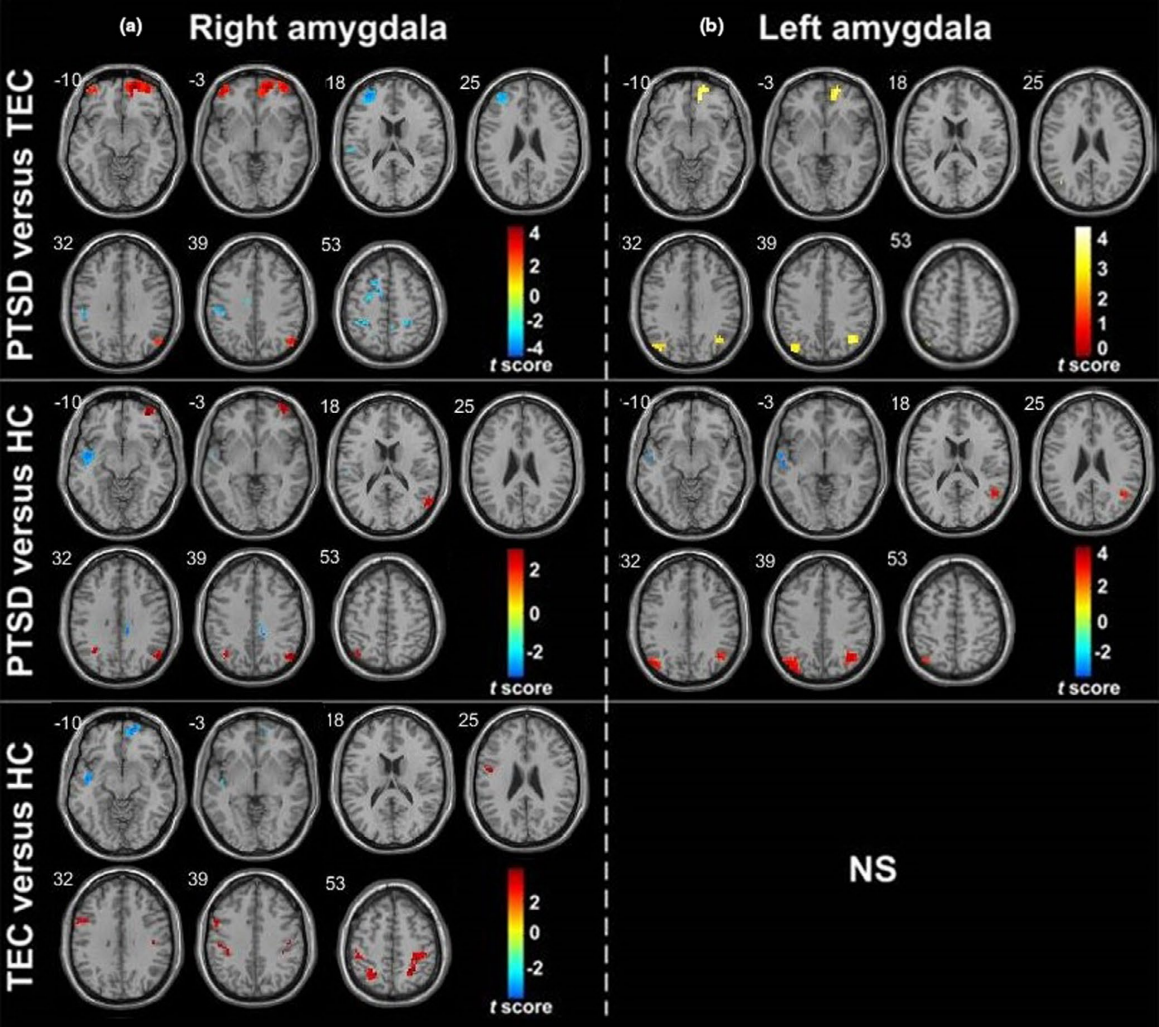
Group differences in whole amygdala resting‐state functional connectivity maps. (a) and (b) represent the group differences in right and left whole amygdala functional connectivity, respectively (*p* < .001, cluster >10, uncorrected). Warm color represents the positive functional connectivity; cold color represents the negative functional connectivity.*Note*: HC, healthy control group; PTSD, post‐traumatic stress disorder group; TEC, trauma‐exposed control group

**Figure 4:**
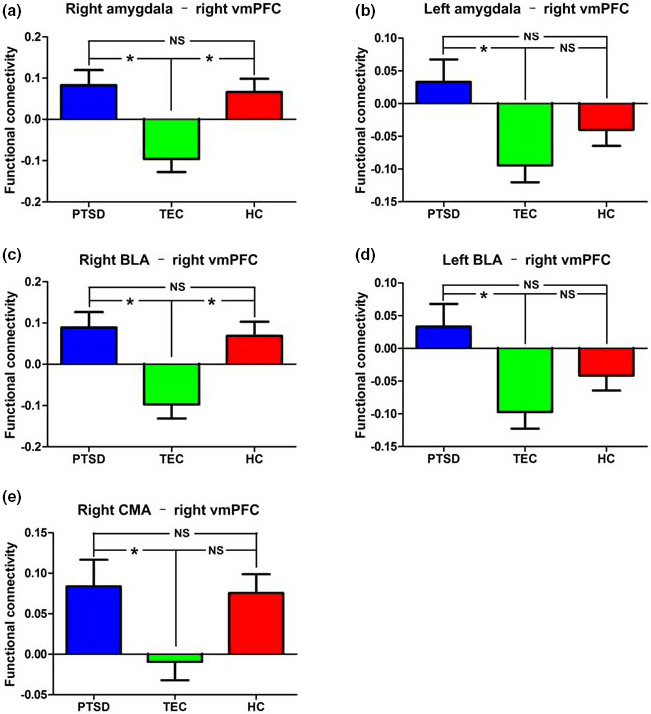
Group differences in whole amygdala and amygdala subregion functional connectivity with the right vmPFC. (a), (b), (c), (d), and (e) represent the group differences in right whole amygdala, left whole amygdala, right BLA, left BLA, and right CMA functional connectivity with the right vmPFC, respectively.*Note*: BLA, basolateral amygdala; CMA, centromedial amygdala; HC, healthy control group; PTSD, post‐traumatic stress disorder group; TEC, trauma‐exposed control group

Compared with the TEC group, the PTSD group showed increased FC between the right amygdala (as a whole) and the right vmPFC, bilateral orbital frontal gyrus (OFC), and right angular gyrus, and decreased FC with the left STG, bilateral PoCG, and anterior MFG. Compared with the HC group, the PTSD group showed increased FC between the right amygdala (as a whole) and the right OFC and bilateral angular gyrus, and decreased FC with the left STG and dACC/paracentral gyrus. Compared with the HC group, the TEC group showed increased FC between the right amygdala (as a whole) and the bilateral PoCG and left paracentral gyrus, and decreased FC with the right vmPFC (Table 2) (Figures 3a and 4a).

**Table 2:** Group differences in right whole amygdala resting‐state functional connectivity

Brain regions	MNI coordinates (mm) (x, y, z)	Voxel number	*t* value^a^
PTSD‐TEC			
Right vmPFC	18, 60, −6	89	4.81
Left OFC	−45, 54, −3	74	3.71
Right OFC	39, 57, −6	77	3.54
Right angular gyrus	48, −66, 42	57	3.35
Left STG	−57, −27, 15	35	−3.17
Left PoCG	−45, −30, 48	167	−3.98
Right PoCG	27, −42, 54	103	−3.66
Left MFG	−30, 45, 21	112	−4.22
PTSD‐HC			
Right OFC	36, 51, −9	43	3.89
Left angular gyrus	−42, −60, 45	42	3.10
Right angular gyrus	51, −66, 39	86	3.42
Left STG	−48, 12, −15	86	−3.68
Right dACC/paracentral gyrus	15, −36, 39	41	−3.75
TEC‐HC			
Left PoCG	−48, −24, 45	56	3.62
Right PoCG	33, −33, 51	106	3.83
Left paracentral gyrus	−9, −36, 66	56	3.46
Right vmPFC	18, 60, −6	40	−3.58

Abbreviations: dACC, dorsal anterior cingulate gyrus; HC, healthy control group; MFG, middle frontal gyrus; MNI, Montreal Neurologic Institute; OFC, orbital frontal gyrus; PoCG, postcentral gyrus; PTSD, post‐traumatic stress disorder group; STG, superior temporal gyrus; TEC, trauma‐exposed control group; vmPFC, ventral media prefrontal cortex.

^a^ Negative sign represents decreased functional connectivity; positive sign represents increased functional connectivity.

Compared with the TEC group, the PTSD group showed increased FC between the left amygdala (as a whole) and the right vmPFC, and bilateral angle gyrus. Compared with the HC group, the PTSD group showed increased FC between the left amygdala (as a whole) and the bilateral angular gyrus, and decreased FC with the left STG. There was no significant difference between the left amygdala FC in the HC group and that of the TEC group (Table 3) (Figures 3b and 4b).

**Table 3:** Group differences in left whole amygdala resting‐state functional connectivity

Brain regions	MNI coordinates (mm)(x, y, z)	Voxel number	*t* value^a^
PTSD‐TEC			
Right vmPFC	15, 54, −6	81	4.51
Left angular gyrus	−39, −75, 36	36	3.11
Right angular gyrus	39, −63, 39	54	3.88
PTSD‐HC			
Left angular gyrus	−42, −72, 39	80	3.46
Right angular gyrus	39, −66, 42	124	4.52
Left STG	−54, 12, −6	72	−3.27

Abbreviations: PTSD, post‐traumatic stress disorder group; TEC, trauma‐exposed control group; HC, healthy control group; MNI, Montreal Neurologic Institute; vmPFC, ventral media prefrontal cortex; STG, superior temporal gyrus; MFG, middle frontal gyrus.

^a^ Negative sign represents decreased functional connectivity; positive sign represents increased functional connectivity.

### 3.4 Group differences in BLA resting‐state FC

**Figure 5:**
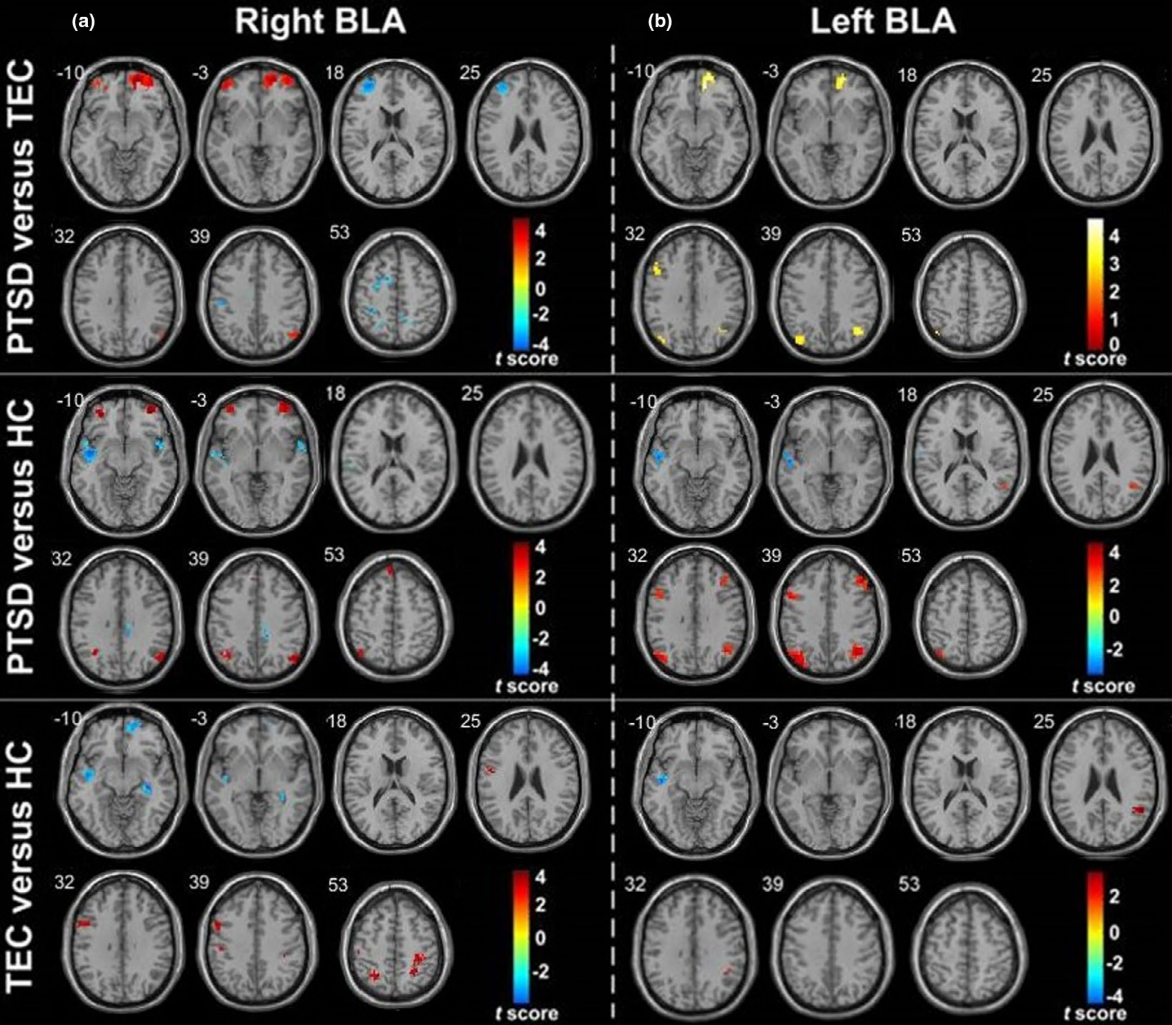
Group differences in BLA resting‐state functional connectivity maps. (a) and (b) represent the group differences in right and left BLA functional connectivity, respectively (*p* < .001, cluster > 10, uncorrected). Warm color represents the positive functional connectivity; cold color represents the negative functional connectivity.*Note*: BLA, basolateral amygdala; HC, healthy control group; PTSD, post‐traumatic stress disorder group; TEC, trauma‐exposed control group

Compared with the TEC group, the PTSD group showed increased FC between the right BLA and the right vmPFC, bilateral OFC and right angle gyrus, and decreased FC with the bilateral PoCG, left SFG, and anterior MFG. Compared with the HC group, the PTSD group showed increased FA between the right BLA and the left vmPFC, bilateral OFC, and angular gyrus, and decreased FC with the bilateral STG and right dACC/paracentral gyrus. Compared with the HC group, the TEC group showed increased FC between the right BLA and the bilateral PoCG, and decreased FC with the right vmPFC, left STG, and right hippocampus (Table 4) (Figures 4c and 5a).

**Table 4:** Group differences in right BLA resting‐state functional connectivity

Brain regions	MNI coordinates (mm) (x, y, z)	Voxel number	*t* value^a^
PTSD‐TEC			
Right vmPFC	18, 57, −6	104	4.76
Left OFC	−45, 54, −3	57	3.54
Right OFC	24, 60, −9	90	3.87
Right angular gyrus	48, −66, 42	38	3.15
Left PoCG	−45, −30, 48	108	−3.78
Right PoCG	27, −39, 51	26	−3.26
Left SFG	−12, 3, 54	68	−3.78
Left MFG	−30, 48, 18	107	−4.07
PTSD‐HC			
Left vmPFC	−6, 48, 45	69	3.28
Left OFC	−30, 51, −12	48	3.88
Right OFC	36, 51, −9	61	3.93
Left angular gyrus	−36, −63, 39	70	3.09
Right angular gyrus	48, −66, 42	92	3.40
Left STG	−42, −3, −12	158	−4.01
Right STG	48, 9, −12	60	−3.39
Right dACC/paracentral gyrus	15, −36, 39	43	−3.79
TEC‐HC			
Left PoCG	−48, −24, 45	48	3.55
Right PoCG	33, −33, 51	107	3.82
Right vmPFC	18, 60, −6	37	−3.55
Left STG	−45, −9, −9	33	−3.61
Right hippocampus	36, −33, −6	43	−3.90

Abbreviations: dACC, dorsal anterior cingulate gyrus; HC, healthy control group; MFG, middle frontal gyrus; MNI, Montreal Neurologic Institute; OFC, orbital frontal gyrus; PoCG, postcentral gyrus; PTSD, post‐traumatic stress disorder group; SFG, superior frontal gyrus; SMA, supplementary motor area; STG, superior temporal gyrus; TEC, trauma‐exposed control group; vmPFC, ventral media prefrontal cortex.

^a^ Negative sign represents decreased functional connectivity; positive sign represents increased functional connectivity.

Compared with the TEC group, the PTSD group showed increased FC between the left BLA and the right vmPFC, bilateral angular gyrus, and left MFG. Compared with the HC group, the PTSD group showed increased FC between the left BLA and the bilateral angular gyrus and MFG, and decreased FC with the left STG. Compared with the HC group, the TEC group showed increased FC between the left BLA and the right supramarginal gyrus/angular gyrus, and decreased FC with the left STG (Table 5) (Figures 4d and 5b).

**Table 5:** Group differences in left BLA resting‐state functional connectivity

Brain regions	MNI coordinates (mm) (x, y, z)	Voxel number	*t* value^a^
PTSD‐TEC			
Right vmPFC	15, 54, −6	91	4.55
Left angular gyrus	−39, −75, 36	58	3.10
Right angular gyrus	39, −63, 39	52	3.77
Left MFG	−51, 18, 36	23	3.08
PTSD‐HC			
Left angular gyrus	−48, −72, 36	94	3.87
Right angular gyrus	39, −66, 42	140	4.62
Left MFG	−51, 15, 36	35	3.38
Right MFG	45, 30, 39	61	3.69
Left STG	−54, 12, −6	87	−3.50
TEC‐HC			
Right supramarginal gyrus/angular gyrus	48, −42, 27	42	3.56
Left STG	−45, −9, −9	41	−4.14

Abbreviations: HC, healthy control group; MFG, middle frontal gyrus; MNI, Montreal Neurologic Institute; PTSD, post‐traumatic stress disorder group; STG, superior temporal gyrus; TEC, trauma‐exposed control group; vmPFC, ventral media prefrontal cortex.

^a^ Negative sign represents decreased functional connectivity; positive sign represents increased functional connectivity.

During the transition from trauma exposure to PTSD, some BLA FC changes are consistent, while other FC changes are opposite, perhaps this reflects the aggravating or modifying factors of the disease process. When compared with the PTSD group and the healthy control group, the TEC group exhibited decreased and increased BLA FC in the vmPFC and PoCG, respectively. Compared with the HC group, the TEC group showed decreased BLA FC in the STG; compared with the healthy control group, the PTSD group also showed decreased BLA FC in the STG (Table 6).

**Table 6:** Group differences tendency in BLA resting‐state functional connectivity

Brain regions	TEC–HC change		PTSD‐HC change		PTSD‐TEC change		Implication
	L.BLA	R.BLA	L.BLA	R.BLA	L.BLA	R.BLA	
Left vmPFC				**↑**			
Right vmPFC		**↓**			**↑**	**↑**	Protective
Left OFC				**↑**		**↑**	
Right OFC				**↑**		**↑**	
Left angular gyrus			**↑**	**↑**	**↑**		
Right angular gyrus			**↑**	**↑**	**↑**	**↑**	
Left PoCG		**↑**				**↓**	Protective
Right PoCG		**↑**				**↓**	Protective
Left STG	**↓**	**↓**	**↓**	**↓**			Aggravated
Right STG				**↓**			
Left MFG			**↑**		**↑**	**↓**	
Right MFG			↑				
Left SFG						**↓**	
Right hippocampus		**↓**					
Right dACC/paracentral gyrus	**↑**			**↓**			

Abbreviations: dACC, dorsal anterior cingulate gyrus; HC, healthy control group; MFG, middle frontal gyrus; OFC, orbital frontal gyrus; PoCG, postcentral gyrus; PTSD, post‐traumatic stress disorder group; SFG, superior frontal gyrus; STG, superior temporal gyrus; TEC, trauma‐exposed control group; vmPFC, ventral media prefrontal cortex.

### 3.5 Group differences in CMA resting‐state FC

**Figure 6:**
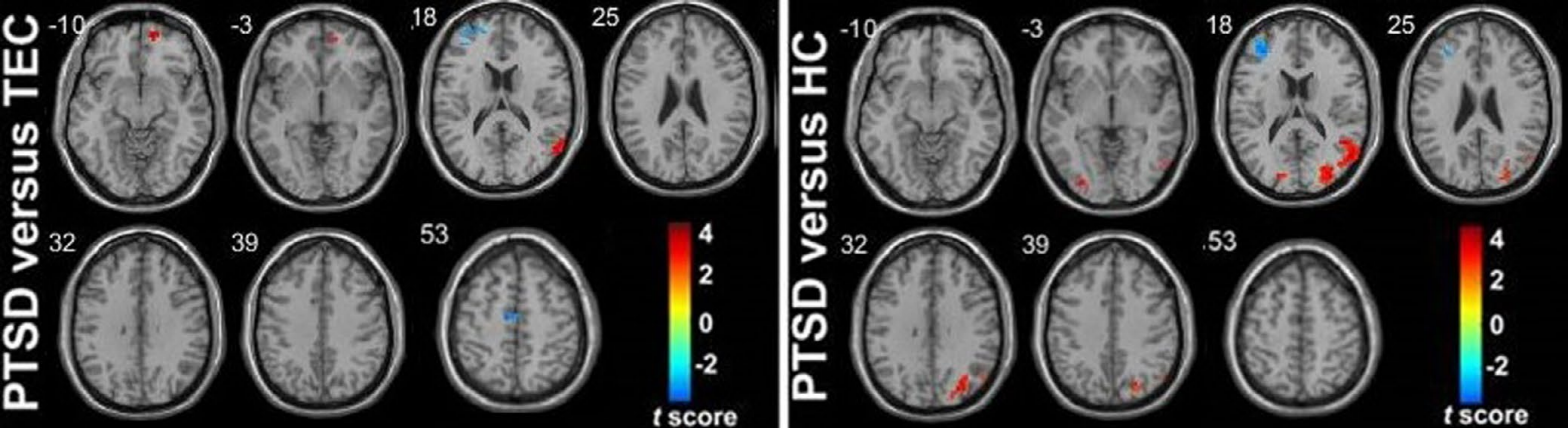
Group differences in right CMA resting‐state functional connectivity maps. It represents the group differences in right CMA functional connectivity (*p* < .001, cluster >10, uncorrected). Warm color represents the positive functional connectivity; cold color represents the negative functional connectivity. Note: CMA, centromedial amygdala; PTSD, post‐traumatic stress disorder group; TEC, trauma‐exposed control group; HC, healthy control group

Compared with the TEC group, the PTSD group showed increased FC between the right CMA and the right vmPFC and right middle temporal gyrus (MTG), and decreased FC with the right PoCG and left MFG. Compared with the HC group, the PTSD group showed increased FC between the right CMA and the right STG and bilateral middle occipital gyrus, and decreased FC with the left anterior MFG. There was no significant difference between the right CMA FC in the HC group and that of the TEC group **(**Figures 4e and 6). There were no significant differences in the left CMA FC of the three groups.

After controlling the data quality by using the scrubbing method (Yan et al., 2013) and additionally including potential nuisance factors as covariates (i.e., age, gender, head motion), all the results remained unchanged.

### 3.6 Correlation analysis results

**Figure 7:**
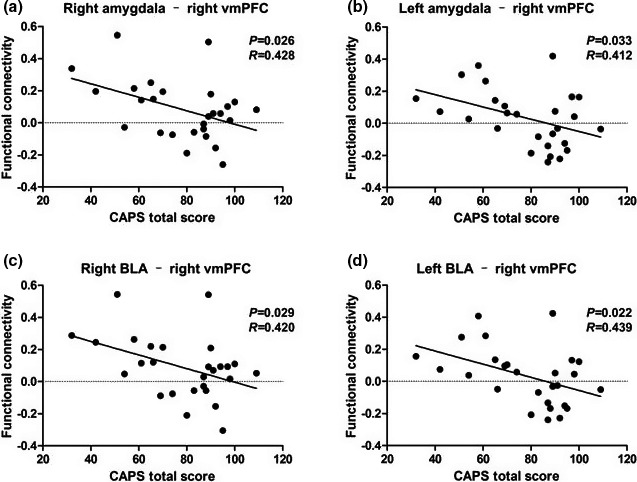
Results of correlation analyses between amygdala and amygdala subregion functional connectivity with vmPFC and total CAPS scores. The total CAPS score is negatively correlated with functional connectivity between the right whole amygdala (a), left whole amygdala (b), right BLA (c), and left BLA (d) with right vmPFC.*Note*: BLA, basolateral amygdala; CAPS, Clinician‐Administered PTSD Scale; vmPFC, ventral media prefrontal cortex

Within the PTSD group, FCs between the right and left amygdala (as a whole) and the right vmPFC were negatively correlated with the total CAPS scores (*p* = .026; 0.033, respectively) **(**Figure 7a,b). The FCs between the right and left BLA and the right vmPFC were also negatively correlated with the total CAPS scores (*p* = .029; 0.022, respectively) (Figure 7c,d). No associations were found between CAPS scores and the FC of the right CMA and right vmPFC.

## 4 DISCUSSION

In this study, we examined FC associated with task‐free spontaneous neural activity in the whole amygdala, the BLA, and the CMA in PTSD, TEC, and HC groups. We demonstrated that the BLA had a stronger connectivity with the PFC and angular gyrus in the PTSD group than in the TEC group. Additionally, compared with the PTSD and the HC groups, the TEC group exhibited decreased and increased BLA FC with the vmPFC and PoCG, respectively. Furthermore, the PTSD group show abnormal FC between the salience network and default‐mode network, as well as the executive control network. We ensured the conclusions using a more stringent voxel‐wise *p* < .001 threshold with cluster size >10 voxels, and the above main findings were still reproduced. Finally, we showed that the FCs between the bilateral amygdala (as a whole) – vmPFC and BLA – vmPFC have a negative correlation with the severity of PTSD. These findings add to our knowledge of altered FC of the amygdala and its subregions, and they provide new insights into the pathophysiology of PTSD.

Similar to previous studies involving untraumatized populations (van Dijk et al., 2007; Uranishi et al., 1999), we observed positive connectivity between the amygdala as a whole and the mPFC, hippocampus, insula, STG, etc., and a negative correlation with the superior frontal gyrus, MFG, and angular gyrus. The results clearly demonstrate the contrasting roles of the BLA and CMA: The BLA connects with cortical regions (mainly in frontotemporal regions) and serves as a stimuli monitor; the CMA connects with subcortical regions such as the striatum to regulate behavioral responses (Tsai et al., 2004) (investigated using resting‐state fMRI seed‐based FC).

We also found differential patterns of FC in these regions in the PTSD and TEC groups. Compared with the TEC group, the PTSD group exhibited increased BLA FC with the vmPFC. The PTSD group also showed a negative correlation with the BLA‐vmPFC in the TEC group when compared with HCs, which is consistent with previous resting‐state fMRI functional connectivity studies (Shin & Liberzon, 2010). Considering the function of vmPFC and its relation to the processing of risk and fear, the inhibition of emotional responses, decision making, and self‐control (Price & Drevets, 2010), we suspect that the relative weakening of amygdala potential and the inhibitory influence of the vmPFC lead to an increased fear response and aberrant emotional regulation in those with PTSD. Differing from previous studies, we found that the negative FC between the BLA and vmPFC in the TEC group was higher than that of the HC group, but there was no significant difference between the PTSD and HC groups. These results may indicate that during the transition from the point of trauma exposure to PTSD, an increased negative correlation between the FC of the BLA and vmPFC may be a protective factor, but in those with PTSD, this positive factor had diminished. Interestingly, we found that the FCs in the PTSD group and the TEC group were negatively correlated with the CAPS scores indicating PTSD severity. Stevens et al. (2013), based on the fear picture task, found that the FC of the amygdala and vmPFC was nonlinearly associated with PTSD severity in all subjects who had experienced trauma. We hypothesized that there may be a similar relationship in the resting state, and the correlation analysis in this study revealed that the PTSD patients with higher symptom scores could lead to seemingly confounding results.

The results of this study also showed that, compared with the PTSD and HC groups, the FC between right BLA and bilateral PoCG was increased in the TEC group (negative FC decreased), which demonstrates that this may be one of the protective factors in the pathogenesis of PTSD. The positive FC between the BLA and the STG was gradually decreased in the HC, TEC, and PTSD groups, which indicates that the trauma could lead to the decrease in FC between the BLA and STG, while PTSD could further aggravate the destruction. Consistent with our results, Sartory et al. (2013), in a meta‐analysis of PTSD brain function studies based on the symptom provocation task, found that the temporal lobe and the posterior central gyrus in patients with PTSD had significantly decreased activation compared with controls. In our study, the FC between the amygdala and the sensory association areas such as the posterior central gyrus and STG relatively deceased; this may be associated with the abnormalities of self‐referential processing in PTSD patients, which directs aberrant resource allocation views of perceptual processing and attention away from nonthreatening sensory information (Guldenmund et al., 2012).

In addition, compared with the TEC and HC groups, the FC between the BLA and bilateral OFC, MFG, and inferior parietal lobule was significantly increased in the PTSD group. The OFC has functional and structural connectivity with the amygdala, which plays an important role in the extinction of conditioned fear, emotion regulation, and the extraction of emotional memory (Bonnelle et al., 2012). PTSD functional (Zhu et al., 2017) and structural (Thomaes et al., 2010) MRI studies have revealed reduced activity and reduced gray matter volume in the OFC of patients with PTSD. In our study, the decreased negative FC between the BLA and OFC in patients with PTSD may be related to the decreased inhibition of the OFC to the amygdala. However, the study found that there was a similar change in brain function in patients with schizophrenia (Anticevic et al., 2014); this suggests that the abnormal connection between the amygdala and the OFC is not specific to PTSD. The bilateral MFG and inferior parietal lobule are the critical brain areas that form the executive network (Seeley et al., 2007; Sylvester et al., 2012). Executive control networks are associated with advanced cognitive functions such as planning, decision making, and working memory (Seeley et al., 2007; Sridharan et al., 2008). In a fMRI study of emotion control tasks, the activation of the MFG in patients with PTSD was attenuated compared with healthy controls (New et al., 2009). A recent meta‐analysis also found abnormal activity of the MFG in PTSD patients at rest (Koch et al., 2016). The amygdala is also an important node of prominent networks, and the salience network is thought to coordinate the balance of the control network and the default network (Sridharan et al., 2008). Therefore, our findings suggest that the salience network and executive control network in PTSD patients have dysfunction in the resting state; this may be related to an impaired cognitive control of emotion.

An important finding of our study is that in the PTSD group, the left anterior MFG had a higher FC with the left BLA compared with that in the TEC and the HC groups. In addition, compared with the HC group, the FC between the BLA and right hippocampus decreased in the TEC groups, but the difference between the PTSD group and the HC group can only be noted through a more relaxed correction (not shown). The FC between the right CMA and the right MTG was significantly higher in the PTSD group than in the HC and TEC groups. The hippocampus, MTG, frontal MFG, and vmPFC are part of the DMN, which is related to introspection, self‐reference processing, and autobiographical memory (Qin & Northoff, 2011; Spreng et al., 2009; Toro et al., 2008). In the present study, FCs between the DMN brain regions and the amygdala subregions were increased or decreased in PTSD patients. Similarly, a recent meta‐analysis of the PTSD task‐state fMRI also found that there is a “separation” phenomenon within the DMN; that is, some of these brain regions are enhanced, and other brain regions have decreased activity (Patel et al., 2012). A number of resting‐state fMRI studies revealed abnormalities in the amygdala and functional regions of the DMN brain areas (such as the posterior cingulate cortex, and hippocampus) in PTSD patients (Bluhm et al., 2009; Koch et al., 2016). Sripada *et al*. used resting‐state fMRI to study veterans and found that the FC between the amygdala and hippocampus was reduced in PTSD patients (Sripada et al., 2012). The FCs between the hippocampus and BLA in the PTSD and TEC groups were not significantly different in this study. However, considering the results of previous studies on hippocampal structure, function, and FC (Acheson et al., 2012; Admon et al., 2009; Felmingham et al., 2009), we think that trauma can cause the alteration of FC between the amygdala and hippocampus, and PTSD may further aggravate the injury.

One limitation in our study was that all subjects in our study were told to have eyes open rather than eyes closed, which might have potential aberrant effects caused by falling asleep. Besides, this study was limited in small sample size and cross‐sectional design. To sum up, the resting‐state FC in the amygdala subregions of PTSD patients, especially in the BLA, is extensively abnormal. The lower FC between the BLA and vmPFC and the higher FC between the BLA and the PoCG may suggest better emotional control and environmental information perception as a protective factor of PTSD. The FC change between the BLA and orbitofrontal gyrus, MFG, inferior parietal lobule, vmPFC, and MTG in PTSD patients may lead to anomalies of dysfunctional integration between the salience network, executive control network, and DMN. Traumatic experiences may cause FC changes in the BLA, the STG, and the hippocampus, and PTSD may aggravate the injury.

## CONFLICT OF INTEREST

There are no competing interests.

## AUTHOR CONTRIBUTIONS

LT performed the analysis and wrote the manuscript. KJ and ZL collected the data and contributed to the discussion. QRF, ZZQ, and XQ contributed to the discussion and manuscript revision. ZY made contributions to the design of the study. LGM revised the manuscript for intellectual content. LT made contributions to the design of the study. CF is the guarantors of this work and, as such, had full access to all the data in the study and takes responsibility for the integrity of the data and the accuracy of the data analysis. All authors read and approved the final manuscript.

### Peer Review

The peer review history for this article is available at https://publons.com/publon/10.1002/brb3.1952.

## Data Availability

The raw/processed data required to reproduce these findings cannot be shared at this time as the data also form part of an ongoing study.
